# Active and passive surveillance for bat lyssaviruses in Italy revealed serological evidence for their circulation in three bat species

**DOI:** 10.1017/S0950268818003072

**Published:** 2018-12-04

**Authors:** S. Leopardi, P. Priori, B. Zecchin, G. Poglayen, K. Trevisiol, D. Lelli, S. Zoppi, M. T. Scicluna, N. D'Avino, E. Schiavon, H. Bourhy, J. Serra-Cobo, F. Mutinelli, D. Scaravelli, P. De Benedictis

**Affiliations:** 1 FAO and National Reference Centre for Rabies, Istituto Zooprofilattico Sperimentale delle Venezie, Viale dell'Università 10, Legnaro (PD), Italy; 2 S.T.E.R.N.A. & Museo Ornitologico ‘F. Foschi’, via Pedrali 12, 47100, Forlì, Italy; 3 Department of Veterinary Medical Science, Alma Mater Studiorum, Via Tolara di Sopra 50, Ozzano dell'Emilia (BO), Italy; 4 Istituto Zooprofilattico Sperimentale delle Venezie, Via Laura Conti 4, Clinical Diagnostic laboratory, Bolzano, Italy; 5 Istituto Zooprofilattico Sperimentale della Lombardia e dell'Emilia Romagna, Via Bianchi 9, Brescia, Italy; 6 Istituto Zooprofilattico del Piemonte, Liguria e Valle d'Aosta (IZSTO), Torino, Italy; 7 Istituto Zooprofilattico Sperimentale delle regioni Lazio e Toscana, Via Appia Nuova 1411, Rome, Italy; 8 Istituto Zooprofilattico Sperimentale dell'Umbria e delle Marche, Via G. Salvemini 1, Perugia, Italy; 9 Istituto Zooprofilattico Sperimentale delle Venezie, Clinical diagnostic laboratory, Viale dell'Università 10, Legnaro (PD), Italy; 10 Institut Pasteur, Unité Dynamique des Lyssavirus et Adaptation à l'Hôte, Paris, France; 11 Universitat de Barcelona, Institut de Recerca de la Biodiveristat, Barcelona, AND Centre de Recerca en Infeccions Víriques, Illes Balears (CRIVIB), Palma de Mallorca, Spain

**Keywords:** Bats, lyssavirus, surveillance

## Abstract

The wide geographical distribution and genetic diversity of bat-associated lyssaviruses (LYSVs) across Europe suggest that similar viruses may also be harboured in Italian insectivorous bats. Indeed, bats were first included within the passive national surveillance programme for rabies in wildlife in the 1980s, while active surveillance has been performed since 2008. The active surveillance strategies implemented allowed us to detect neutralizing antibodies directed towards *European bat 1 lyssavirus* in six out of the nine maternity colonies object of the study across the whole country. Seropositive bats were *Myotis myotis*, *M. blythii* and *Tadarida teniotis.* On the contrary, the virus was neither detected through passive nor active surveillance, suggesting that fatal neurological infection is rare also in seropositive colonies. Although the number of tested samples has steadily increased in recent years, submission turned out to be rather sporadic and did not include carcasses from bat species that account for the majority of LYSVs cases in Europe, such as *Eptesicus serotinus*, *M. daubentonii*, *M. dasycneme* and *M. nattereri.* A closer collaboration with bat handlers is therefore mandatory to improve passive surveillance and decrypt the significance of serological data obtained up to now.

## Short report

Rabies is a major zoonosis accounting for an estimated 61 000 yearly deaths, mostly in Asia and Africa [1]. Despite the fact that the large majority of rabies fatalities are caused by dog-associated rabies virus (RABV), other pathogens belonging to the genus *Lyssavirus* can cause in humans a disease clinically indistinguishable from rabies [2]. Currently, the International Committee on Taxonomy of Viruses (ICTV) recognises 16 species among the genus *Lyssavirus*, plus two related viruses whose taxonomy remains undetermined [3]. Besides *Mokola lyssavirus* and *Ikoma lyssavirus*, all species have been found in bats worldwide. Among these, six are reported to circulate in Europe, namely *European bat 1 lyssavirus* (EBLV-1), *European bat 2 lyssavirus* (EBLV-2), *Bokeloh bat lyssavirus* (BBLV), *West Caucasian bat lyssavirus* (WCBV), *Lleida bat lyssavirus* (LLEBV) and the newly described *Kotalahti bat lyssavirus* (KBLV) [2]. These viruses have been mostly reported in specific bat hosts, including *Eptesicus serotinus* and *E. isabellinus* for EBLV-1, *Myotis daubentonii* and *M. dasycneme* for EBLV-2, *M. nattereri* for BBLV, *M. schreibersii* for WCBV and LLEBV, and *M. brandtii* for KBLV [2].

However, serological studies across Europe suggest that other species might be involved in the ecology of EBLVs [4]. Spillover to non-flying mammals is reported for EBLV-1, although perpetuation in secondary hosts has never occurred [5].

Despite the large viral diversity found in chiropters, a lyssavirus (LYSV) case occurring in a bat does not have the same public and veterinary health or economic impact as a rabies case in non-flying mammals. Thus, the rabies status of a country currently refers to the epidemiology of classical rabies only [1]. However, as bat LYSVs have been associated with lethal clinical rabies in humans, it is advisable that national surveillance programmes are implemented throughout Europe to gain more detailed information about their geographical spread and epidemiology. As for classical rabies, priority should be given to passive surveillance on index animals, providing a higher chance for virus detection. On the other hand, live sampling of bats is essential to further investigate the dynamics of these pathogens within their natural hosts. However, as all bat species are protected within Europe, the potential impact on bat conservation must be considered while designing and undertaking surveillance programmes.

To date, no rabies cases in bats have been notified in Italy. Bats have been included within the passive surveillance programme for rabies in wildlife since the 1980s, with 154 individuals from 10 different species analysed between 1986 and 1993, also including seven serotine bats [6]; further surveillance on carcasses was not reported until its reinforcement in 2006. On the other hand, active surveillance was first implemented in 2008. In this study, we describe 11 years of surveillance for bat LYSVs in Italy between 2006 and 2017 and report the detection of sero-positivity anti EBLV-1 in six bat colonies across the country, often confirmed along consecutive years. We discuss plausible explanations for failing to detect viral infection in the Italian bats and highlight the importance of further strengthening passive surveillance activities.

### Passive surveillance

In Italy, the national passive surveillance network for rabies in wildlife is based on the examination of dead, sick or injured animals that may have been in contact with humans or domestic animals, in compliance with the national regulation on animal health and welfare D.P.R. 08/02/1954 n. 320. Eight laboratories belonging to the national health network of the 10 existing Istituti Zooprofilattici Sperimentali (II.ZZ.SS.) are accredited for official rabies testing in animals, with performances checked through an annual proficiency trial organised by the National Reference Centre for Rabies (NRCR) hosted at the Istituto Zooprofilattico Sperimentale delle Venezie (IZSVe, Legnaro, Padua) in north-eastern Italy. Each laboratory provides quarterly reports to the NRCR. For the purpose of the present study, the participating laboratories were asked to provide more specific information in addition to the data routinely submitted, such as the bat species, method adopted for host classification, collection date and location, name of the collector and history of contacts with humans and/or domestic animals.

Among the eight laboratories of the network, five claimed to have received bat carcasses and samples between 2006 and 2017. When applicable, the fluorescent antibody test (FAT) was always performed as gold standard technique for the diagnosis of rabies [1, 7]. In addition, three laboratories performed further confirmatory tests, including either virus isolation attempts (either the mouse inoculation test (MIT; *n* = 2) or the rapid tissue culture infection test (RTCIT; *n* = 58)) and the one-step RT-PCR [8], since 2009 and 2012, respectively. Concerning virus isolation, no MITs have been performed on bat samples since 2010. Starting from 2015 and 2017, respectively, two of the laboratories sent all the collected bat samples (either the whole carcass or the head) to the NRCR for testing.

In total, Italian laboratories received 296 bat carcasses between 2006 and 2017. Among these, 115 were screened for the presence of LYSVs’ antigen using FAT, 50% of which were further analysed by RT-PCR (*n* = 58, 50.4%) and/or virus isolation attempts (*n* = 60, 52.2%). Between 2015 and 2017, 176 additional brain specimens were received and tested using molecular techniques only, as the poor quality of the samples prevented any attempt of performing FAT (Supplementary Table S1). Results of molecular analyses were considered reliable based on the amplification of the housekeeping gene 18s (primers available upon request), which proved successful in all but five samples, which were excluded from analyses. Thus, 291 bats were screened for LYSV in Italy between 2006 and 2017, using FAT, virus isolation and/or molecular methods (Supplementary Table S1).

**Fig. 1.**
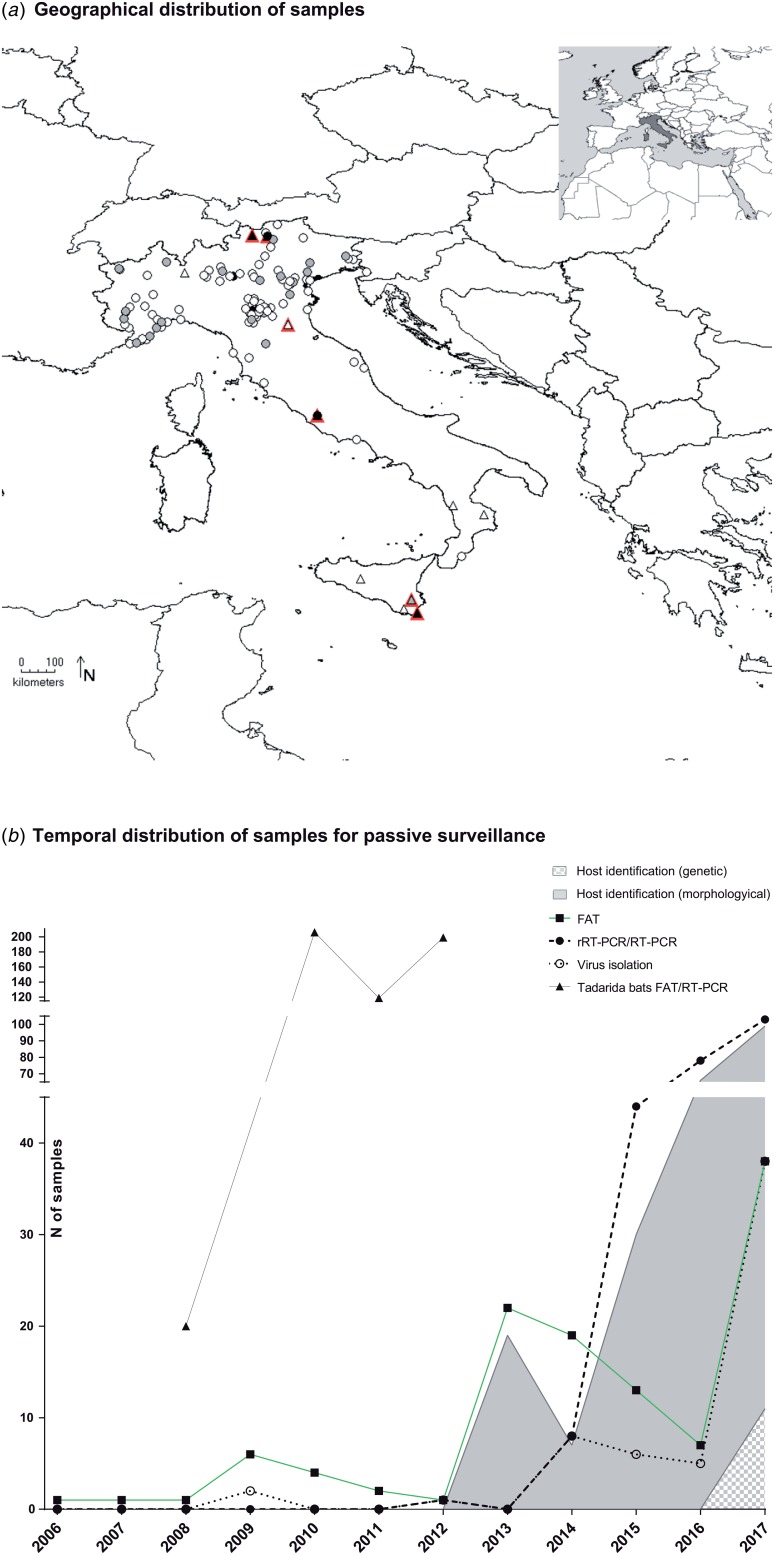
Active and passive surveillance for lyssaviruses circulating in Italian bats (2006–2017). (a) Geographical location of bat carcasses (circles) and colonies (triangles) subject to passive and active surveillance. Colours from white to black indicate an increased sampling effort. In particular, locations are indicated with white, grey and black circles if one, 2–10 or >10 bats were received from the same area. Similarly, triangles are white, grey or black, respectively, for one, two and over three sampling campaigns in the same colony. Sero-positive colonies are shown in red. (b) Passive surveillance. Number of brain samples analysed in the frame of passive surveillance and year of analysis. Black squares, black circles and empty circles indicate number of FAT as gold standard technique, of molecular methods and of virus isolation attempts, respectively. Virus isolation was performed using either the MIT or the RTCIT between 2006 and 2011, while only the RTCIT has been used since 2012. Grey and grey-square areas under the curves indicate morphological or genetic host identification, respectively. Dead bats collected from the maternity colony of *T. teniotis* are shown separately (black triangles). These latter were all analysed using both FAT and RT-PCR.

The number of samples analysed per year ranged between 1 and 103, with a constant increase registered since 2013 (Fig. 1b). Most samples were collected from northern Italy (*n* = 282, 96.9%), fewer from central (*n* = 7, 2.4%) and southern regions (*n* = 2, 0.7%), none from the islands (Fig. 1a, Supplementary Table S1). Mostly, the animals were submitted by wild animal rehabilitation centres (*n* = 208, 71.5%) or through the public local veterinary services (*n* = 65, 25.4%). In 5.1% of cases, samples (*n* = 15) were collected in bat roosts during active surveillance activities. It was referred that 23 out of the 291 animals submitted for diagnosis had had contacts with humans or domestic cats, accounting for 16 human bites.

None of the bat species most frequently infected with LYSVs in Europe (including *E. serotinus*, *M. daubentonii*, *M. dasycneme*, *M. nattereri*, *M. brandtii* or *M. schreibersii*) had been submitted to any of the Italian laboratories during the reporting period, with the exception of one *M. schreibersii* individual in a time span of 11 years. The most represented host species were *Pipistrellus kuhlii* (*n* = 96, 32.4%), *Hypsugo savii* (*n* = 82, 27.7%) and *M. myotis* (*n* = 14, 4.7%), while 28.9% of bats (*n* = 84) were not identified (Supplementary Table S1). Critically, most of the unclassified specimens belonged to suckling pipistrelle bats, readily distinguishable from either serotine, myotis or miniopterus species. The determination of the host species has steadily increased, with an average of 80% of samples identified since 2013. Morphological keys or expert opinion were mostly used until 2017, when genetic confirmation through the partial sequencing of the cytochrome b (primers available upon request) was introduced, leading so far to the identification of 12 of 103 samples (11% of those submitted in 2017) (Fig. 1b).

All samples tested negative for the presence of LYSVs either using FAT or through virus isolation or RT-PCR (Supplementary Table S1).

### Active surveillance

Live sampling of bats for rabies surveillance was first introduced in Italy in 2008.

The methods used during fieldwork differ according to the bat species and the characteristics of the roost. The animals were captured using either mist nets positioned at the cave entrance or through hand nets, and then placed in cotton bags so that they could calm down for 5–10 min. Around 4–6 *µ*l of blood/g of body weight was drawn from the uropatageal or the brachial vein under physical restraint, using capillary tubes or 0.3 ml insulin syringe. Bats were given water before being released to prevent dehydration. Salivary samples were obtained through active biting of dry paediatric tracheal swabs. All samples were kept at +4 °C in the field, and then stored at −20 or −80 °C before serological or virological analyses, respectively. Carcasses in a good state of preservation were always collected and analysed for LYSV antigen, virus or RNA detection using the methods described above. Species identification was also performed with the collaboration of bat handlers.

Blood samples were pre-treated with 12% PEG for 24 h to maximise immunoglobulin extraction from coagula, after which PBS with antibiotics was added at 50:50. Sera were pre-diluted 1:30 (final dilution 1:67 raising at 1:201 at the first dilution well when incubated with cells and virus) and were analysed for the presence of antibodies against LYSVs using a modified Rapid Fluorescent Focus Inhibition Test (RFFIT) [9] and EBLV-1a or EBLV-2 as challenge viruses. Samples were considered positive against the challenge virus when inhibiting the virus at 50% at the first dilution analysed (1:201), corresponding to Log *D*_50_/ml ⩾2.3, as determined by using the Reed–Muench formula.

For positive bat species, we analysed the proportion of seropositive individuals with respect to the month of sampling, sex, age and sampling area (northern Italy/Sicily) using the Pearson's *χ*^2^ or the Fisher's exact test depending on the frequency. Only sampling years and colonies showing positive cases were included in the statistical analyses.

RNA was extracted from salivary samples, SNC homogenates and/or blood clots using the Nucleospin RNA kit (Macherey-Nagel, Duren, Germany) and the presence of LYSV was assessed through molecular testing using a protocol slightly modified from Wakeley *et al*. [10] using SYBR(®) Green (Applied Biosystems, Foster City, CA, USA) (2008 only) or a single-step RT-PCR [8], as described elsewhere.

Over the 7 years of the study, 10 bat roosts were screened across Italy for the circulation of LYSVs (Fig. 1a, Supplementary Table S1). Among these, six were underground sites (caves/mines) and four were located within human settlements. Active campaigns were performed in the southern (two roosts in Calabria) and northern regions (four roosts in Emilia-Romagna, Lombardy and South Tyrol) and in the island of Sicily (four roosts) (Fig. 1a, Supplementary Table S1). Most sites hosted maternity colonies with occasional detection of isolated males. Six bat species were analysed, including *E. serotinus*, *M. schreibersii*, *M. blythii*, *M. capaccini*, *M. myotis* and *Rhinolophus ferrumequinum.* Most roosts hosted mixed colonies, with the exception of two aggregations of *R. ferrumequinum* and *E. serotinus*, respectively (Supplementary Table S1). Four colonies were sampled more than once. Among these, one colony was sampled over 2 years and three colonies during 3 years; in addition, two roosts were visited twice over a single reproductive season. The lesser mouse-eared bat (*M. blythii*) and the greater mouse-eared bat (*M. myotis*) were sampled more widely in nine of 10 and eight of 10 locations during 16 of 19 and 14 of 19 campaigns, respectively (Supplementary Table S2).

Both blood and salivary samples were collected at least once from all sites: out of 19 sampling campaigns, blood was collected in 18 occasions and saliva in 15. For each site and at each sampling time, we collected 2–65 blood samples (mean 21) and 5–74 throat swabs (mean 20). Among blood samples, 89.9% provided sufficient material for serological analyses (Supplementary Table S1). Unfortunately, the poor quality of all blood samples from the only maternity colony of serotine bats under study prevented us from performing any serological analysis in this host species.

Antibodies against EBLV-1 have been regularly recorded every year since 2009, while no positive cases were found in 2008 (Supplementary Table S1). Eight sampling campaigns showed at least one positive individual. Among nine sites visited for serological investigations, five mixed maternity colonies of sibling greater and lesser mouse-eared bats tested sero-positive at least once. Despite co-roosting with *M. capaccini* (*n* = 2), *M. schreibersii* (*n* = 3) and/or *R. ferrumequinum* (*n* = 2) being detected in some location, antibodies against EBLV-1 were found exclusively in mouse-eared bats. The percentage of sero-positivity within colonies varied between 1.5% and 73.3%, with mean values of 31.5% and 29%, respectively in *M. myotis* and *M. blythii* (Supplementary Table S2). Antibodies titres were low, ranging from 2.3 to 3.04 (mean 2.5) logD_50_. Of note, six animals from three colonies showed virus inhibition under the cut-off value.

Among four colonies screened longitudinally, a single positive case was detected in colony Sicily 3 in 2011, but not in the two campaigns of 2008, nor in 2012. Interestingly, the maternity colony of mouse-eared bats South Tyrol 1, located in the roof of an active church, was investigated twice in the same year, showing weaning of antibodies at the end of the season. However, most of the colony had already left the summer roost at the time of fieldwork, reducing sample size to 12 individuals, which is predicted to detect a positive case (CI 95%) only for sero-prevalence ⩾22% (Supplementary Table S1).

Indeed, the month of sampling was found to have statistical influence on the likelihood for sero-positivity (*P* = 0.0001), with an increased sero-prevalence starting from June. Similarly, the year of sampling and the sex of the animal had a significant correlation with sero-positivity (*P* < 0.0001; *P* = 0.0048), while the age and the area of sampling (northern *vs.* southern regions) had none (*P* = 0.146; *P* = 0.478).

Sixty-five serum samples from myotis bats were further screened for the presence of antibodies against EBLV-2, testing all negative samples (Supplementary Table S1).

No viral RNA was detected from any of the salivary swabs analysed throughout the study, including samples from serotine bats collected from a small colony located within a school. In addition, 136 blood clots collected in 2008 from five colonies in Southern Italy were screened for the presence of LYSV RNA, when no seropositive cases were detected. All samples tested negative (Supplementary Table S1).

### Investigation of an urban colony of free-tailed bats during a mass mortality event

Between 2008 and 2012, a mass mortality event interested an urban colony of free-tailed bats (*Tadarida teniotis*) located in a crowded area of Rome, on the sixth floor between two blocks of flats. In particular, young animals before fledging were affected by a severe deformation of the joints and bones and died each year between July and August.

During the event, 544 brain samples and 254 hearts were submitted to the NRCR for rabies testing. This included 20, 206, 119 and 199 brains (Fig. 1b) paired with 20, 90, 91 and 53 blood clots from the heart cavity collected in 2008, 2010, 2011 and 2012, respectively (Supplementary Table S1). All samples came from animals in their first year of age.

Brain samples were homogenised and analysed for the presence of a LYSV infection, as described above. Blood clots were treated and analysed for the presence of specific neutralizing antibodies, as described above.

All brain samples tested negative for LYSV infection. On the other hand, the colony showed an epidemic curve of exposure to EBLV-1, with no antibodies in 2008, rising up to 21.8% of positivity in 2010, decreasing in 2011 (6.6%) until complete waning in 2012 (Supplementary Table S1). As for myotis bats, we performed analyses of cross-neutralisation in 19 positive samples, with no inhibition of EBLV-2.

## Discussion

Overall, results from the present study provide evidence for the presence and natural circulation of LYSVs in Italian bats. Indeed, active surveillance highlighted the presence of specific antibodies in at least three bat species across the country, namely *M. myotis*, *M. blythii* and *T. teniotis.* In all the cases, antibodies specifically neutralised EBLV-1, confirming similar findings which had previously been notified in Spain [9]. Cross-reactivity shown towards EBLV-2 was negligible, differently to what observed in other studies in Daubenton's bats (*M. daubentonii*) between EBLV-2 and EBLV-1 [11]. Despite the large variability between colonies and sampling times, our data support on average a higher percentage of sero-positivity compared with other studies performed on several bat species for both EBLV-1 and EBLV-2 in other European countries [11, 5, 12]; however, our result is in line with previous findings from Spain in both *M. myotis* and *T. teniotis* [9, 13, 14]. Although these differences might represent regional or species-specific changes in virus dynamics, the comparison between different studies is strongly challenged by differences in the sampling scheme, the neutralisation test used and the cut-off value used [12].

Interestingly, the serological positivity of free-tailed bats was detected during a mass mortality multiannual event. However, the fact that no specific antigen/RNA was detected in any carcass analysed from the colony suggested that death had not been directly caused by LYSV infection.

Indeed, this is in line with the results observed in other species, showing exposure to EBLV-1 with no relevant impact on mortality rate [9, 15]. Interestingly, all animals analysed during the first and the last year of sampling tested sero-negative. Unfortunately, the relocation after 2012 of the Roman colony prevented further sampling to test whether the population was subject to a single epidemic wave or if there is a cyclic temporal fluctuation of bat infections and seroconversion, as already suggested for this bat species [14]. A similar fluctuation in the level of sero-prevalence was detected in myotis bats throughout the study period. These data would further support the existence of cyclic waves within bat populations [9, 14, 15]. However, it is crucial to mention how the possible intra-annual seasonal pattern might confound the comparison of inter-annual data collected in different moments of the season. Indeed, sero-prevalence is statistically influenced by the month of sampling in our dataset, which confirms the impact of ecological factors operating at species and community levels on the sero-prevalence against EBLV-1, as suggested for the Spanish colonies [4].

Critically, three of six positive maternity colonies were located within human settlements, including two churches and a block of flats, suggesting possible human exposure to infected bats. However, disease surveillance in bat carcasses from all the colonies failed to detect LYSVs, supporting very low virus prevalence especially in the Roman colony, for which we screened a high amount of carcasses.

In contrast with the results obtained in other countries in Europe, no virus was detected in the framework of passive surveillance in Italy. However, authors have to admit that the number of analyses in Italy was significantly low throughout the whole study period with no hosts identified as either *E. serotinus*, *M. daubentonii*, *M. dasycneme* or *M. nattereri*, which account for the majority of LYSV cases in Europe [2]. In addition, the study highlights the need for further improving and harmonizing diagnostic flowcharts adopted at a national level for LYSV detection. As the diagnostic sensitivity of classical techniques for RABV might turn out as suboptimal for bat-LYSVs [16], since 2016 the ability of each laboratory to diagnose a bat-LYSVs infection has been assessed on a yearly basis with satisfying results. In this regard, it would be critical to extend the use of a validated pan-LYSV RT-PCR protocol at a national level to ensure a reliable diagnosis in these hosts. Indeed, the use of molecular methods allowed us to screen also samples of poor quality or available in very small quantities, both very common circumstances in the surveillance of small wild mammals.

Within this context, the NRCR is endeavouring to implement a surveillance network that includes all the laboratories involved in bat surveillance within the territory, with the aim of harmonizing the diagnostic methods adopted in the Italian territory for LYSV detection. The cooperation between health institutions and bat conservationists has also been enhanced through *ad hoc* awareness initiatives and the provision of guidelines for a safe handling of bats. The synergic implementation of a more solid surveillance system able to uncover the geographical boundaries of LYSV circulation is paramount to quantify its actual risk. Critically, the International Union for Conservation of Nature (IUCN) has stated that 22 of 33 bat species present in Italy are declining in the territory and are classified as near-threatened or threatened. Thus, the involvement of bat experts for disease surveillance, communication and, eventually, for the development of mitigation strategies, is strongly encouraged to combine public health with bat conservation.

## Supplementary material

For supplementary material accompanying this paper visit https://doi.org/10.1017/S0950268818003072. click here to view supplementary material
