# Supplementary material for: Active and passive surveillance for bat lyssaviruses in Italy revealed serological evidence for their circulation in three bat species
Source: Epidemiol Infect. 2018 Dec 4;147:e63. doi: 10.1017/S0950268818003072 (PMC6518613; doi:10.1017/S0950268818003072)
Supplement: Supplementary file 1 [file S0950268818003072sup.zip › S0950268818003072sup001.docx]

Epidemiology and Infection

Active and passive surveillance for bat lyssaviruses in Italy revealed serological evidence for their circulation in three bat species

S. Leopardi, P. Priori, B. Zecchin, G. Poglayen, K. Trevisiol, D. Lelli, S. Zoppi, M.T. Scicluna, N. D’Avino, E. Schiavon, H. Bourhy, J. Serra-Cobo, F. Mutinelli, D. Scaravelli, P. De Benedictis

Supplementary Table 1. Bat samples submitted and analysed for LYSV surveillance in Italy since 2006

| Type of surveillance | Geographic distribution^1^ | Type of sample | Diagnostic method^2^ | Positive/tested per analysis | Sampling time^3^ | Host species^4^ | Serology^5^ positive/tested per species | Virology^6^ positive/tested per species |
| --- | --- | --- | --- | --- | --- | --- | --- | --- |
| Passive | N: 282  C: 7  S: 2 | Brain | FAT  RTCIT  MIT  RT-PCR | 0/115 +176 ns^7^  0/58  0/2  0/234 +5 ns | 2006 – 2017 | Undetermined  *H. savii*  *M. myotis*  *M. mystacinus*  *M. nattereri*  *M. schreibersii*  *N. noctula*  *P. auritus*  *P. kuhlii*  *P. nathusii*  *P. pipistrellus*  *R. ferrumequinum*  *R. hipposideros* |  | 0/84  0/82 +2 ns  0/14  0/3  0/1  0/1  0/5  0/6  0/96 +3 ns  0/1  0/1  0/1  0/1 |

| Mass mortality events | Lazio (C) | Brain | FAT + RT-PCR | 0/20 | 2008 | *T. teniotis* | 24/254 +4 ns | 0/544 |
| --- | --- | --- | --- | --- | --- | --- | --- | --- |
|  |  |  |  | 0/206 | 2010 |  |  |  |
|  |  |  |  | 0/119 | 2011 |  |  |  |
|  |  |  |  | 0/199 | 2012 |  |  |  |
|  |  | Blood | RFFIT EBLV-1 | 0/19 | 2008 |  |  |  |
|  |  |  |  | 19/87 | 2010 |  |  |  |
|  |  |  |  | 5/91 | 2011 |  |  |  |
|  |  |  |  | 0/53 | 2012 |  |  |  |
|  |  |  | RFFIT EBLV-2 | 0/19 | 2010 |  |  |  |
| Active | Calabria 1 (S) | Blood | RFFIT EBLV-1 | 0/5 | 2008 Jul | *R. ferrumequinum* | 0/5 | 0/5 |
|  |  |  | rRT-PCR | 0/1 |  |  |  |  |
|  |  | Saliva | rRT-PCR | 0/5 |  |  |  |  |
|  | Calabria 2 (S) | Blood | RFFIT EBLV-1 | 0/41 | 2008 Sep | *M. blythii*  *M. myotis*  *M. schreibersii* | 0/2  0/26  0/13 | 0/2  0/26  0/15 |
|  |  |  | rRT-PCR | 0/36 |  |  |  |  |
|  |  | Saliva | rRT-PCR | 0/38 |  |  |  |  |
|  | Emilia-Romagna (N) | Blood | RFFIT EBLV-1 | 1/2 | 2013 Sep | *M. blythii*  *M. schreibersii*  *R. ferrumequinum* | 1/1  0/1 | 0/1  0/14 |
|  |  | Saliva | RT-PCR | 0/15 |  |  |  |  |
|  | Lombardy (N) | Blood | RFFIT EBLV-1 | 8 ns | 2012 Jul | *E. serotinus* |  | 0/9 |
|  |  | Saliva | RT-PCR | 0/9 |  |  |  |  |

|  | Sicily 1 (I) | Blood | RFFIT EBLV-1 | 0/38 | 2008 Jul | *M. blythii*  *M. capaccini*  *M. myotis*  *M. schreibersii* | 0/3  0/8  0/20  0/7 | 0/3  0/8  0/20  0/7 |
| --- | --- | --- | --- | --- | --- | --- | --- | --- |
|  |  |  | rRT-PCR | 0/38 |  |  |  |  |
|  |  | Saliva | rRT-PCR | 0/38 |  |  |  |  |
|  | Sicily 2 (I) | Blood | RFFIT EBLV-1 | 3/16 | 2009 Jun | *M. blythii*  *M. capaccini*  *M. myotis*  *M. schreibersii* | 1/4  3/22 | 0/4  0/4  0/22  0/9 |
|  |  |  |  | 1/10 | 2011 Jun |  |  |  |
|  |  | Saliva | RT-PCR | 0/16 | 2009 Jun |  |  |  |
|  |  |  |  | 0/23 | 2011 Jun |  |  |  |
|  | Sicily 3 (I) | Blood | RFFIT EBLV-1 | 0/23 | 2008 Jun | *M. schreibersii*  *M. blythii*  *M. capaccini*  *M. myotis* | 0/24 +8 ns  0/4 +3 ns  0/1  1/38 + 10 ns | 0/26  0/7  0/1  0/48 |
|  |  |  |  | 0/25 | 2008 Sep |  |  |  |
|  |  |  |  | 1/15 | 2011 Jun |  |  |  |
|  |  |  |  | 0/4 +21 ns | 2012 Sep |  |  |  |
|  |  |  | rRT-PCR | 0/23 | 2008 Jun |  |  |  |
|  |  |  |  | 0/24 | 2008 Sep |  |  |  |
|  |  | Saliva | rRT-PCR | 0/23 | 2008 Jun |  |  |  |
|  |  |  |  | 0/25 | 2008 Sep |  |  |  |
|  |  |  |  | 0/15 | 2011 Jun |  |  |  |
|  |  |  |  | 0/19 | 2012 Sep |  |  |  |

|  | Sicily 4 (I) | Blood | RFFIT EBLV-1 | 0/18 | 2008 Jun | *M. blythii*  *M. myotis*  *R. ferrumequinum* | 0/2  0/2  0/14 | 0/2  0/2  0/10 |
| --- | --- | --- | --- | --- | --- | --- | --- | --- |
|  |  |  | rRT-PCR | 0/14 |  |  |  |  |
|  |  | Saliva | rRT-PCR | 0/14 |  |  |  |  |
|  | South Tyrol 1 (N) | Blood | RFFIT EBLV-1 | 2/5 +5 ns | 2012 Jul | *M. blythii*  *M. myotis* | 0/7 +1 ns  9/89 +6 ns | 0/88  0/11 |
|  |  |  |  | 6/14 +2 ns | 2013 Sep |  |  |  |
|  |  |  |  | 1/65 | 2015 May |  |  |  |
|  |  |  |  | 0/12 | 2015 Sep |  |  |  |
|  |  |  | RFFIT EBLV-2 | 0/65 | 2015 May |  |  |  |
|  |  | Saliva | RT-PCR | 0/11 | 2012 Jul |  |  |  |
|  |  |  |  | 0/14 | 2013 Sep |  |  |  |
|  |  |  |  | 0/74 | 2015 May |  |  |  |
|  | South Tyrol 2 (N) | Blood | RFFIT EBLV-1 | 11/15 | 2013 Sep | *M. blythii*  *M. myotis* | 9/29  3/8 | 0/17  0/4 |
|  |  |  |  | 1/22 | 2015 Sep |  |  |  |
|  |  | Saliva | RT-PCR | 0/6 | 2012 Sep |  |  |  |
|  |  |  |  | 0/15 | 2013 Sep |  |  |  |

^1^ N, C, S and I indicate Northern, Central, Southern Italy and Islands, respectively, therefore including the following regions: N: Emilia-Romagna, Friuli-Venezia Giulia, Liguria, Lombardy, Piedmont, Trentino-South Tyrol, Aosta Valley, Veneto; C: Lazio, the Marches, Tuscany, Umbria; S: Abruzzo, Basilicata, Calabria, Campania, Molise, Apulia; I: Sardinia, Sicily.

^2^Acronyms indicates following methods: FAT, fluorescent antibody test; RTCIT, rapid tissue culture infection test; MIT, mouse inoculation test; RT-PCR, retro-transcriptase polymerase chain reaction; rRT-PCR, real time retro-transcriptase polymerase chain reaction; RFFIT EBLV-1/2, rapid fluorescent focus inhibition test against European bat lyssavirus 1 or 2

^3^Information on the sampling time include the year only for the surveillance during mass mortality event, while the month of collection is specified, when available, for the active surveillance. Acronyms indicates the following months: Jun, June; Jul, July; Sep, September.

^4^Bat species analysed (full names): *Hypsugo savii, Miniopterus schreibersii, Myotis blythii, Myotis capaccini, Myotis myotis, Myotis mystacinus, Myotis nattereri, Nyctalus noctula, Plecotus auritus, Rhinolophus ferrumequinum, Rhinolophus hipposideros, Pipistrellus kuhlii, Pipistrellus nathusii, Pipistrellus pipistrellus, Tadarida teniotis.*

^5^Data summarized in this section includes results from serological analyses (RFFIT).

^6^Data summarized in this section includes results from classical (FAT) and molecular (rRT-PCR/PCR) virology.

^7^ns: non suitable for analyses. Samples have been considered suitable for FAT based on quantity and quality of the brain material; molecular methods have been attempted for FAT unsuitable samples. Samples have been considered suitable for molecular analyses if tested as positive for the presence of the housekeeping gene 18s. Blood samples have been considered as suitable for serological examinations based on their volumes and absence of cytotoxic effect.
