# Supplementary material for: Active and passive surveillance for bat lyssaviruses in Italy revealed serological evidence for their circulation in three bat species
Source: Epidemiol Infect. 2018 Dec 4;147:e63. doi: 10.1017/S0950268818003072 (PMC6518613; doi:10.1017/S0950268818003072)
Supplement: Supplementary file 1 [file S0950268818003072sup.zip › S0950268818003072sup002.docx]

Epidemiology and Infection

Active and passive surveillance for bat lyssaviruses in Italy revealed serological evidence for their circulation in three bat species

S. Leopardi, P. Priori, B. Zecchin, G. Poglayen, K. Trevisiol, D. Lelli, S. Zoppi, M.T. Scicluna, N. D’Avino, E. Schiavon, H. Bourhy, J. Serra-Cobo, F. Mutinelli, D. Scaravelli, P. De Benedictis

Supplementary Table 2. Active surveillance: results from serological analyses against EBLV-1 for species, location and sampling time (2008-2015).

| Colony | Sampling time | Positive/analysed samples (percentage %) | | | | |
| --- | --- | --- | --- | --- | --- | --- |
|  |  | *M. schreibersii* | *M. blythii* | *M. capaccini* | *M. myotis* | *R. ferrumequinum* |
| Calabria 1 | 2008 Jul |  |  |  |  | 0/5 (0) |
| Calabria 2 | 2008 Sep | 0/13 (0) | 0/2 (0) |  | 0/26 (0) |  |
| Emilia-Romagna | 2013 Sep |  | 1/1 (100) |  |  | 0/1 (0) |
| Sicily 1 | 2008 Jul | 0/7 (0) | 0/3 (0) | 0/8 (0) | 0/20 (0) |  |
| Sicily 2 | 2009 Jun |  |  |  | 3/16 (18.7) |  |
|  | 2011 Jun |  | 1/4 (25) |  | 0/6 (0) |  |
| Sicily 3 | 2008 Jun | 0/8 (0) |  |  | 0/15 (0) |  |
|  | 2008 Sep | 0/15 (0) | 0/3 (0) | 0/1 (0) | 0/6 (0) |  |
|  | 2011 Jun |  |  |  | 1/15 (6.7) |  |
|  | 2012 Sep | 0/1 | 0/1 |  | 0/2 |  |
| Sicily 4 | 2008 Jun |  | 0/2 (0) |  | 0/2 (0) | 0/10 (0) |
| South Tyrol 1 | 2012 Jul |  | 0/3 (0) |  | 2/2 (100) |  |
|  | 2013 Sep |  | 0/2 (0) |  | 6/12 (50) |  |
|  | 2015 May |  | 0/2 (0) |  | 1/63 (1.6) |  |
|  | 2015 Sep |  |  |  | 0/12 (0) |  |
| South Tyrol 2 | 2013 Sep |  | 8/11 (72.7) |  | 3/4 (75) |  |
|  | 2015 Sep |  | 1/18 (5.5) |  | 0/4 (0) |  |

^1^Bat species analysed (full names): *Miniopterus schreibersii, Myotis blythii, Myotis capaccini, Myotis myotis, Rhinolophus ferrumequinum.*
